# Synergistic Effect of Aminated Sodium Alginate Composite Material on Selective Adsorption of Sb: Experimental and Density Functional Theory Study

**DOI:** 10.3390/gels11121019

**Published:** 2025-12-18

**Authors:** Lan Yang, Pingshu Wang, Xin Sun, Kai Li, Meijie Ren, Fansong Liu, Fawei Tang, Ping Ning, Yimin Huang

**Affiliations:** 1 Faculty of Environmental Science and Engineering, Kunming University of Science and Technology, Kunming 650500, China; 2 Hangzhou Yanqu Information Technology Co., Ltd., Hangzhou 310003, China

**Keywords:** Sb(III) adsorption, aminated sodium alginate gel beads, DFT calculation, H bonding, complexation

## Abstract

Pollution by Sb, which is widely used in industry and agriculture, poses serious threats to ecosystems. This study demonstrates, for the first time, that sodium alginate (ALG) modified by polyethyleneimine (PEI) has good adsorption capacity for Sb(III) (the theoretical maximum adsorption capacity was 978 mg/g, and the actual maximum adsorption capacity was 743 mg/g) and can retain 90–98% of the initial removal rate after eight cycles of reuse. The inorganic ions and humic acid in Sb(III)-containing wastewater do not affect the adsorption capacity of PEI/ALG within a certain pH range. However, it was also found that the adsorption was interfered with by Sb(III) precipitation, phosphate ions, and some coexisting cations/metalloids such as Ni, Cd, Pb, and As under higher pH conditions, and the recovery rate of antimony in the desorption process needs to be further improved. Density functional theory calculations reveal that the -OH, -COOH, -NH_2_, -NH-, and -N= in PEI/ALG show strong binding with Sb (−56.85, −28.39, −17.98, −25.76, and −17.98 kcal/mol, respectively), enabling these functional groups to easily form stable composite structures with Sb(III). This characteristic enables PEI/ALG to selectively adsorb Sb(III) under certain conditions. Combining these findings with the characterization analysis results indicates that the mechanism of PEI/ALG adsorption of Sb(III) is mainly the formation of H bonds and coordination between -OH, -COOH, and Sb(III). The selective adsorption mechanism of PEI/ALG for Sb(III) has not been investigated previously, and our research results indicate the high potential of this approach.

## 1. Introduction

Sb is a metalloid element widely used in military equipment, flame retardants, lead-acid batteries, and other raw materials [1]. Sb is highly toxic to all life forms and has been classified as a strong carcinogen by the World Health Organization (WHO). The acute toxicity of Sb(III) is 10 times higher than that of Sb(V) [2]. The Sb concentration in the wastewater produced during the mining and smelting process of Sb ore can reach as high as 100–7000 mg/L [3]. In the natural environment, Sb degradation is difficult, making Sb extremely prone to continuous accumulation and causing serious environmental pollution problems. The WHO has established a guideline of 5 μg/L as the maximum acceptable concentration of Sb in drinking water [4]. Over the past 20 years, the pollution caused by Sb mine wastewater in China has affected the safety of drinking water for nearly 9 million people. Therefore, effective treatment of Sb-containing wastewater is urgently needed [1].

The US Geological Survey in 2023 reported that the global supply–demand gap for Sb is expected to reach 18,000 t by 2030. The United States, the European Union, and China, as well as other countries, have classified Sb as a critical strategic mineral. Therefore, the recovery of Sb from Sb-containing wastewater is an effective method for Sb pollution control [1]. Currently, control of Sb(III) pollution mainly involves methods such as adsorption [5], membrane filtration [6], coagulation [7], and ion exchange [8]. Among these treatment technologies, adsorption is considered a promising method for Sb pollution control because of its simplicity, strong regeneration capability, and ability to recover Sb [9]. The removal and recovery of Sb by adsorption are limited by several factors. First, based on the characteristics of Sb-containing wastewater, in the pH range of 5.0–9.0, the adsorbent should have high selective adsorption capacity for Sb in the presence of CO_3_^2−^, PO_4_^3−^, Cl^−^, Ca^2+^, Mg^2+^, Cd^2+^, Ni^2+^, and dissolved organic matter (DOM). Therefore, new adsorbents with high selectivity for adsorbing Sb(III) are urgently needed.

Previous studies have shown that nitrogen, oxygen, and sulfur atoms have strong coordination ability with Sb(III) ions. Wang et al. have reported that hyperbranched polyamide-functionalized sodium alginate (ALG) can selectively adsorb Sb(III), but its maximum adsorption capacity is only 35.57 mg/g [10]. Huang et al. reported that amino-functionalized lignin-based porous biochar exhibits highly selective adsorption performance for Sb(III) (371.7 mg/g). Under pH 5 conditions, the coexisting anions, cations, and humic acid exert a relatively minor interference effect on the adsorption of Sb(III) by the adsorbent [11]. The aforementioned studies indicate that ALG and materials rich in amino groups have the potential to selectively adsorb Sb(III). Thus, ALG materials modified with polyethyleneimine (PEI) may have excellent potential for the highly selective adsorption of Sb(III) owing to the abundant amine groups in PEI. At present, PEI/ALG composites have been widely used for the adsorption and removal of heavy metal ions such as Cr and Pb. However, there have been no reports on the selective adsorption mechanism of Sb(III) by PEI/ALG composites.

The unique feature of ALG lies in the highly dense distribution of carboxyl (-COOH) and hydroxyl (-OH) functional groups in its molecular chain [12,13,14]. The introduction of PEI can enhance the three-dimensional network structure of ALG and introduce high-density amino groups (-NH_2_, -NH-, and =N-). The main functional groups in the PEI/ALG composite include hydroxyl (-OH), carboxyl (-COOH), amino (-NH_2_), imine (-NH-), and imine (=N-) groups [15,16]. When Sb(III) coexists with other common ions (e.g., CO_3_^2−^, PO_4_^3−^, Cl^−^, Ca^2+^, Mg^2+^, Cd^2+^, and Ni^2+^) and DOM in wastewater, the strength of the interaction between these functional groups and Sb(III) (usually manifested as the binding energy) is the key factor that determines the selectivity of the PEI/ALG composite for Sb(III). The larger the negative binding energy between the functional group and Sb(III) is (i.e., the higher the absolute value), the easier its binding with Sb(III) is [11], and the higher the selective adsorption ability of the functional groups for Sb(III) is. The method used to prepare the PEI/ALG composite determines the distribution characteristics and densities of carboxyl, hydroxyl, and amino groups. Therefore, the modification method has a significant effect on the adsorption capacity of the composite for Sb(III).

Alginate and polyethyleneimine composites have been studied in the field of heavy metal adsorption. However, there is still a lack of systematic explanation for the deep mechanism of Sb(III) adsorption. In this study, we explored adsorbent preparation methods that enhance the adsorption capacity and stability of adsorbents by investigating the crosslinking of ALG and PEI. The selective capacity of PEI/ALG for Sb(III) adsorption, the effects of the environmental factors on the adsorption process of Sb(III) on PEI/ALG, and the regenerative utilization capability of PEI/ALG for Sb(III) were studied. In this study, the adsorption mechanism of PEI/ALG on Sb(III) was deeply revealed by combining density functional theory (DFT) calculation and system characterization for the first time, and the role of functional groups in selective adsorption was clarified, thus filling the research gap of this material in Sb(III) adsorption, especially in microscopic mechanism. The related work provides a solid theoretical basis for the efficient and selective adsorption of Sb(III) by PEI/ALG, and also provides a new idea for the development of functional materials for antimony pollution control.

## 2. Results and Discussion

### 2.1. Characterization and Analysis of PEI/ALG

#### 2.1.1. SEM Analysis

**Figure 1:**
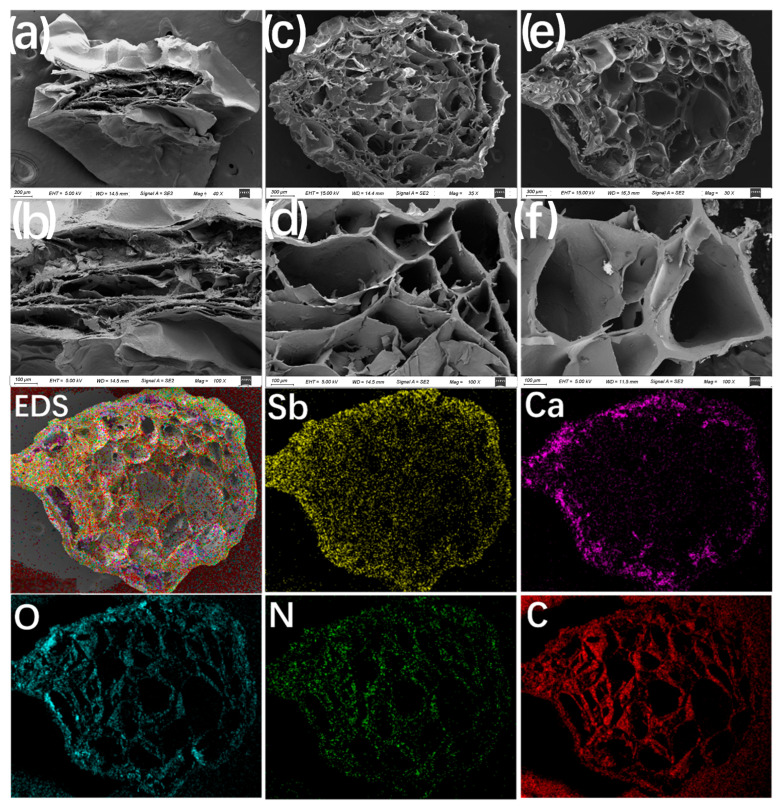
(**a**,**b**) SEM images of ALG; SEM images before (**c**,**d**) and after PEI/ALG adsorption of Sb(III) (**e**,**f**); EDS elemental mapping.

The surface characteristics of ALG and PEI/ALG are depicted in Figure 1. The cross-section of the ALG gel beads shows narrow internal channels and dense voids (Figure 1a,b). After the introduction of PEI, PEI/ALG forms a 3D network with a loose porous structure (Figure 1c,d). These porous structures expose more active sites and shorten the transmission distance between molecules [17]. After PEI/ALG adsorbs Sb, the gel beads maintain a 3D network structure, and the internal porous channels are not broken (Figure 1e), indicating that the structure is stable. The pore wall is thickened because of the adsorption of Sb (Figure 1f). The EDS diagram of PEI/ALG shows that O, N, C, and Ca are uniformly distributed on the gel beads, indicating that PEI is uniformly grafted onto the ALG beads. The adsorbed Sb is also evenly distributed on the surface and inside the gel beads, indicating that the 3D network structure of PEI/ALG is conducive to Sb adsorption.

The Brunauer–Emmett–Teller results indicate that the average pore diameters of ALG and PEI/ALG are 55 and 260 nm, respectively, further demonstrating that the introduction of PEI enhances the porous structure of ALG. After Sb adsorption, the average pore diameter of PEI/ALG decreases to 51 nm. This reduction is attributed to the occupation of the internal channels by a large amount of adsorbed Sb, which also accounts for the thickening of the pore walls (Figure 1f).

#### 2.1.2. XRD Analysis

**Figure 2:**
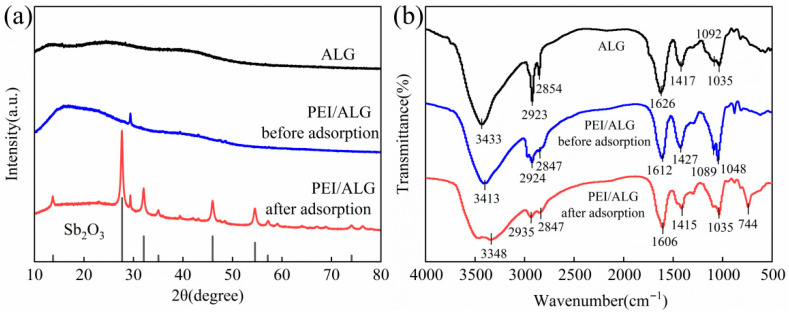
(**a**) XRD patterns before and after PEI/ALG adsorption of Sb(III); (**b**) FTIR spectra of ALG and PEI/ALG before and after adsorption of Sb(III) (peaks are indicated in the image).

The characteristic peak of ALG appears at approximately 30º (Figure 2a). After loading with PEI, the characteristic peaks of ALG shift and their intensities increase. A strong and broad amorphous peak of PEI appears at 20º, proving that PEI has been successfully loaded onto ALG. PEI contains numerous amino functional groups in its molecular structure, which can produce strong H bonds with ALG molecules, making PEI evenly distributed in ALG gel beads [18]. After Sb(III) adsorption by the PEI/ALG gel beads, characteristic diffraction peaks of Sb_2_O_3_ appear at 2θ = 13.74º, 27.70º, 32.08º, 35.04º, 45.99º, 54.55º, 57.13º, and 74.06º, indicating that the PEI/ALG gel beads have successfully adsorbed Sb(III).

#### 2.1.3. FTIR Analysis

For ALG beads (Figure 2b), the peak representing the O-H stretching vibration appears at 3433 cm^−1^. The peaks representing the C-H stretching and bending vibrations appear at 2923 and 2854 cm^−1^, respectively. The asymmetric and symmetric vibration peaks of the COOH group appear at 1626 and 1417 cm^−1^, respectively [18]. The stretching vibrations of the C-O chains, which can be observed in both -COO− and C-O-H configurations, are located at 1092 cm^−1^ and 1035 cm^−1^ respectively [19,20,21]. After the introduction of PEI, the peak at 3433 cm^−1^ is shifted to 3413 cm^−1^ and becomes wider, mainly owing to the overlap of the O-H groups in ALG and N-H groups in PEI [19,21]. In addition, due to the overlap of the -NH_2_ and -NH- of PEI with the -COOH groups, the asymmetric and symmetric vibration peaks of the -COOH groups shift from 1626 and 1417 cm^−1^, to 1612 and 1427 respectively, and the peak intensities increase [21,22].

After the PEI/ALG adsorbs Sb(III) (Figure 2b), the infrared spectrum undergoes significant changes: the O-H peak shifts from 3413 cm^−1^ to 3348 cm^−1^, and the COO^-^ peaks shift from 1612 cm^−1^ to 1606 cm^−1^ and from 1427 cm^−1^ to 1415 cm^−1^. Furthermore, the emergence of a new band at 744 cm^−1^ can be ascribed to the Sb-O-Sb stretching vibration [12]. After the desorption of PEI/ALG, the peak representing Sb-O-Sb disappears, indicating that the adsorbed Sb has been successfully desorbed.

#### 2.1.4. XPS Analysis

**Figure 3:**
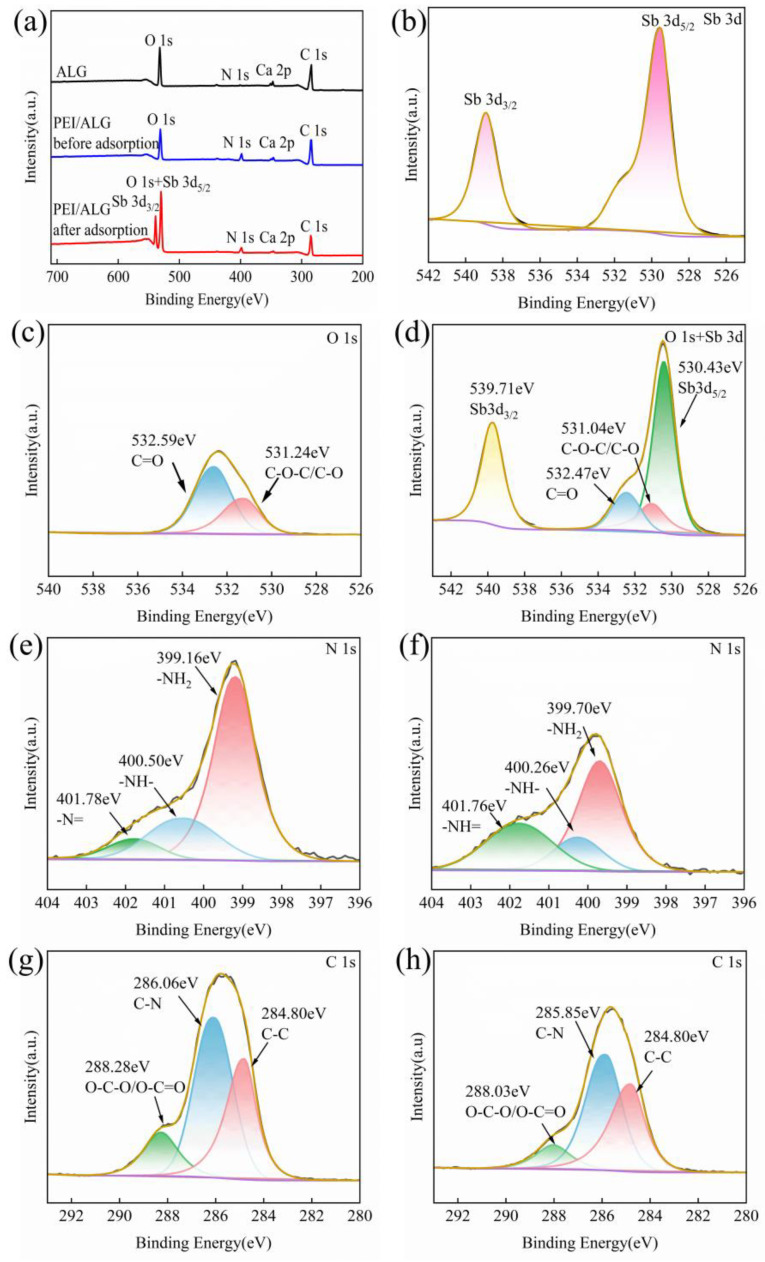
XPS spectra of (**a**) ALG and PEI/ALG and (**b**) Sb 3d; O 1S XPS spectra (**c**) before and (**d**) after ad-sorption; N 1S XPS spectra (**e**) before and (**f**) after adsorption; C 1S XPS spectra (**g**) before and (**h**) after adsorption.

Figure 3a presents the comprehensive XPS results for the ALG and PEI/ALG gel beads before and after Sb(III) adsorption. After the addition of PEI, the N content in PEI/ALG increases from 1.47% to 9.36%. The reason for the increase in the N content is that PEI is a polyamine containing multiple amine groups, and the introduction of PEI increases the N content in PEI/ALG, indicating that PEI has been successfully grafted onto ALG. After the reaction between PEI/ALG and Sb, the absorption peaks of C, O, and N in PEI/ALG change significantly, and an Sb peak appears, indicating that PEI/ALG gel beads have successfully adsorbed Sb by the C, O, and N groups. The high-resolution Sb 3d XPS spectrum (Figure 3b) clearly shows characteristic peaks of Sb 3d_5/2_ and Sb 3d_3/2_ representing Sb(III) at 529.52 and 538.9 eV.

Figure 3c,d present the high-resolution O 1s spectra of PEI/ALG before and after Sb(III) adsorption, respectively. The peaks of PEI/ALG at 532.59 and 531.24 eV before adsorption correspond to the vibrations of C=O and C-O-C/C-O from the -COOH groups, respectively [21,23,24]. After Sb(III) adsorption, the O1s peak overlaps with the Sb3d_5/2_ and Sb3d_3/2_ peaks, and the C=O peak proportion diminishes from 64.13% to 12.34%, whereas the C-O-C/C-O peak proportion declines from 35.87% to 12.22%. Simultaneously, the peak position shifts, indicating that C=O and C-O-C/C-O participate in the adsorption of Sb(III) by PEI/ALG through the formation of C-O-Sb bonds [12]. -OH and -COOH participate in Sb(III) removal through H bonding and coordination effects [11].

Figure 3e,f show the high-resolution N 1s spectra of PEI/ALG. The peaks of PEI/ALG at 399.16, 400.5, and 401.78 eV correspond to the -NH_2_, -NH-, and -N= groups, respectively. After Sb(III) adsorption, the positions of these three peaks change owing to the complexation of -NH_2_, -NH-, and -N= with Sb. Based on the results of DFT calculations (Section 2.1.5), -NH- preferentially complexes with Sb, followed by -NH_2_ and -N= complexation with Sb.

Figure 3g,h show the high-resolution C 1s spectra of PEI/ALG. The peaks of PEI/ALG at 284.8, 286.06, and 288.28 eV are attributed to the C-C (34.78%), C-N (51.43%), and O-C-O/O-C=O (13.8%) groups, respectively [25,26,27]. After PEI/ALG adsorbed Sb(III), the proportion of the O-C-O/O-C=O peak decreased from 13.8% to 10.69%, which is due to the H bonding between Sb(OH)_3(aq)_ and the -COOH ligand. Consequently, C-O-Sb is formed. The shift in the C-N peak is caused by complexation between NH- and Sb(OH)_3(aq)_ [11].

#### 2.1.5. DFT Calculations

**Figure 4:**
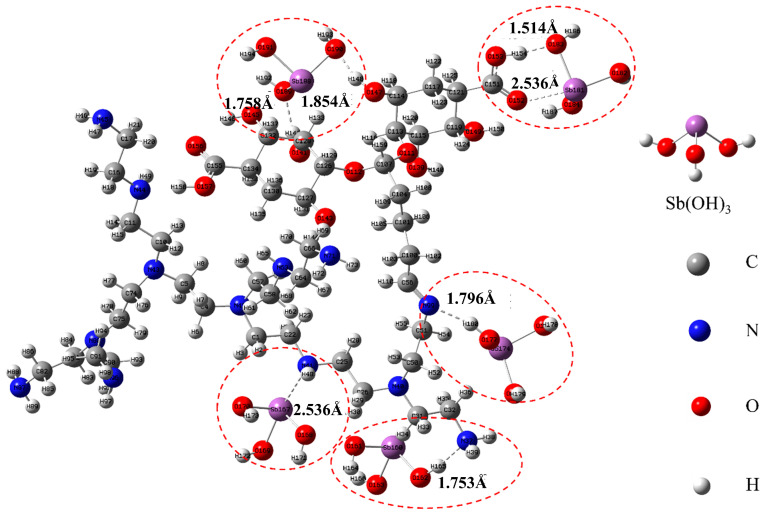
Optimized geometric structure of gel beads.

The mechanism of Sb(III) removal by PEI/ALG was investigated further by conducting DFT calculations (Figure 4). Sb(OH)_3_ is the main Sb form at pH 6, and a structural model of PEI/ALG binding to Sb(OH)_3(aq)_ was constructed using the -OH, -COOH, and amino (-NH_2_, -NH-, and-N=) groups on PEI/ALG as the binding sites. After model optimization, the binding energies of these complexes were calculated. The results show that -OH, -COOH, -NH_2_, -NH-, and -N= form stable complexes with Sb(OH)_3(aq)_, with the calculated binding energies of −23.76, −16.19, −13.87, −17.65, and −10.26 kcal/mol, respectively (Table 1). The more negative the binding energy of the calculated composite molecule, the more easily the composite molecule can bind and the more stable the composite structure. Therefore, the reaction pathway order of Sb with the PEI/ALG functional groups is -OH, -NH-, -COOH, -NH_2_, and -N=. Sb(OH)_3(aq)_ preferentially reacts with -OH and -NH- on PEI/ALG and then reacts with -COOH, -NH_2_, and -N=. The O-H and Sb-O bond lengths in the -COOH-Sb complex on PEI/ALG are 1.514 and 2.536 Å, respectively, while the H-O and H-O bond lengths in the OH-Sb complex are 1.758 and 1.854 Å, respectively; the N-H bond length in the NH_2_-Sb complex is 1.753 Å, that of N-Sb in the -NH-Sb complex is 2.536 Å, and that of N-H in the -N=Sb complex is 1.796 Å. Based on the DFT calculations and characterization analysis, the most likely pathway for PEI/ALG adsorption of Sb is through the complexation of the -NH-, -NH_2_, and -N= groups with Sb (OH)_3(aq)_, with -OH and -COOH participating in Sb(III) adsorption through H bonding and coordination interaction [11].

It should be noted that the DFT calculation in this study is based on a simplified molecular model, which does not consider the spatial conformation of the actual polymer chain and the synergistic effect between functional groups. Therefore, the binding energy and bond length data obtained are mainly used for qualitative comparison of the relative trend of each functional group, rather than quantitative description of the real adsorption system.

### 2.2. Adsorption Mechanism

By combining FTIR, XPS and DFT calculations, this study systematically elucidated the synergistic mechanism of various functional groups in the adsorption of Sb(III) by PEI/ALG. The FTIR results show that the O-H and COO^-^ peaks shift to lower frequencies, and the intensities of the C-H and C-O stretching vibration peaks decrease. Simultaneously, a new peak representing Sb-O-Sb appears at 744 cm^−1^, indicating that O-H and COO^-^ participate in Sb adsorption. The above experimental results are in good agreement with the density functional theory calculations. Theoretical simulation shows that the binding energies of-OH and -COOH functional groups with Sb(OH)_3(aq)_ are −23.76 and −16.19 kcal/mol, respectively, and stable coordination bonds (such as O-Sb in -COOH-Sb) are formed in the optimized geometric configuration. This indicates that the oxygen functional groups play an important role in the adsorption process by forming stable coordination bonds. The XPS results also show that after Sb(III) adsorption, the O1s peak of PEI/ALG overlaps with the Sb3d_5/2_ and Sb3d_3/2_ peaks and, -COOH groups formed C-O-Sb complexes with Sb, leading to a reduction in the intensity of the O-C-O/O-C=O peak [28]. The above results indicate that OH and COOH groups interact with Sb through hydrogen bonding and the coordination effect.

**Figure 5:**
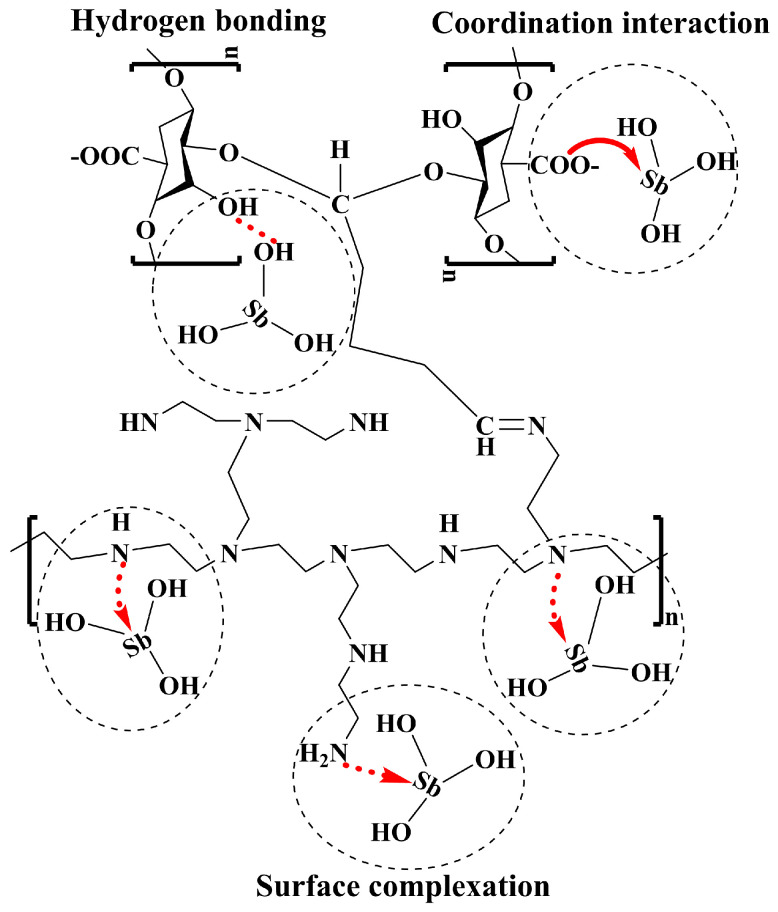
Mechanism of Sb adsorption by PEI/ALG.

The peak positions of the -NH_2_, -NH-, and -N= groups change after Sb adsorption, indicating that the amine group forms a complex with Sb. Based on DFT calculations, the-NH_2_, -NH-and-N = groups show a strong complexation trend with Sb(III), and the binding energy of -NH-Sb(III) is relatively lower, indicating that it may form a more stable coordination structure. The XRD results show that some Sb is adsorbed on the surface of PEI/ALG in the form of Sb_2_O_3_, which may be due to the fact that PEI/ALG promotes the formation of Sb(OH)_3_ solid, and the sample drying process may lead to the conversion of Sb(OH)_3(s)_ to Sb_2_O_3_ (Figure 5).

**Figure 6:**
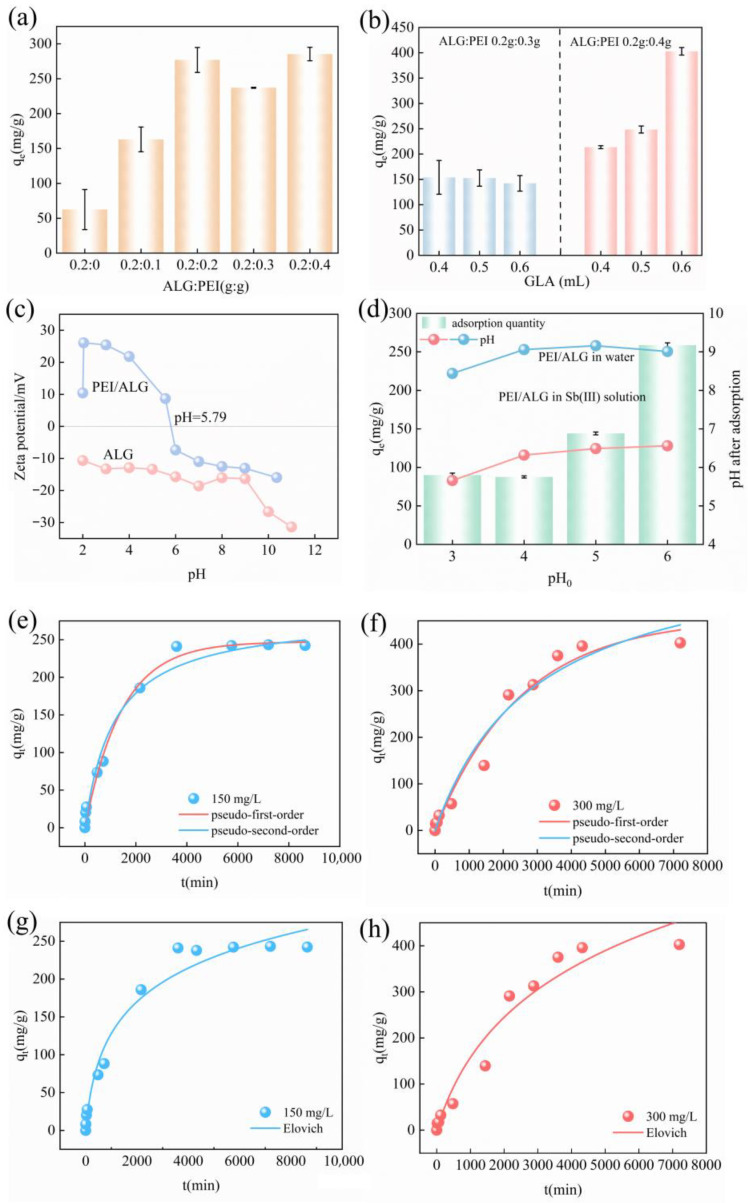
Influence of (**a**) ALG/PEI ratio and (**b**) GLA content on the adsorption capacity of the adsorbents; (**c**) zeta potential of PEI/ALG gel spheres at different pH values; (**d**) pH changes before and after the addition of ultrapure water and Sb solution; (**e**,**f**) adsorption kinetics of Sb(III) on PEI/ALG gel spheres; (**g**,**h**) adsorption kinetics of Sb(III) according to the Elovich model.

In light of the aforementioned results, the mechanism of PEI/ALG adsorption of Sb is as follows (Figure 6): (1) -OH and -COOH react with Sb through H bonding and coordination effects; (2) NH_2_, -NH-, and -N= react with Sb through complexation; (3) some Sb(OH)_3(s)_ is converted into Sb_2_O_3_ and adsorbed on the surface of PEI/ALG.

### 2.3. Influence of Different Component Proportions on the Adsorption Performance of Gel Beads

To investigate the influence of different proportions of PEI and ALG, as well as the effects of variations in the crosslinking agent contents, on the adsorption capacity of gel beads, a series of experimental results were compared. As shown in Figure 6a, the adsorption capacity of ALG gel beads was only 58 ± 6 mg/g. When ALG was introduced into PEI, with the amount of added PEI increasing from 0.1 g to 0.3 g, the adsorption of Sb on PEI/ALG-beads increases from 160.7 ± 17 mg/g to 234.0 ± 0.7 mg/g, which is 4.8 times greater than that of ALG, demonstrating that the incorporation of PEI enhances the adsorption capacity of ALG. When the addition of PEI is increased from 0.3 g to 0.4 g, although the amount of Sb adsorbed on PEI/ALG increases to 279.1 ± 7.2 mg/g, the gel bead structure of PEI/ALG is unstable and prone to cracking. This is because when the amount of added PEI is increased to 0.4 g, the content of the crosslinking agent is no longer sufficient to crosslink PEI and ALG, resulting in some PEI was not cross-linked with sodium alginate.

In view of the results mentioned above, we adjusted the crosslinking agent content when the amount of PEI was 0.3 and 0.4 g. As can be observed from Figure 6b, when the amount of PEI is 0.3 g, the amount of Sb adsorbed on PEI/ALG slightly decreases as the crosslinking agent content increases from 0.4 mL to 0.6 mL. This result indicates that 0.6 mL of crosslinking agent is excessive for crosslinking between PEI and ALG, and the crosslinking agent occupies the adsorption sites on gel beads. When 0.4 g of PEI is added, the amount of Sb adsorbed on PEI/ALG increases to 402 ± 7.4 mg/g as the crosslinking agent content increases to 0.6 mL, and the structure of PEI/ALG remains stable.

The compressive test results (Figure S1) further reveal the change in mechanical properties of the material: the stress–strain curves of all gel balls show two regions: the initial linear elastic region with a stable slope, indicating reversible deformation; in the subsequent plastic deformation zone, the stress decreases sharply with the increase in strain to the peak, showing brittle characteristics [29]. The compressive strength of pure ALG gel balls at 40% strain was only 0.046 MPa. With the introduction of PEI, the compressive strength of the gel balls was significantly improved, which was attributed to the interpenetrating dual network structure formed by PEI and ALG, which enhanced the mechanical stability of the material. In addition, when the additional amount of PEI was fixed at 0.4 g, the compressive strength of the gel balls was further improved as the crosslinking agent GLA increased from 0.4 mL to 0.6 mL. This further shows that the appropriate amount of crosslinking agent is very important for the formation of a complete and stable crosslinking network; if the cross-linking agent is insufficient, it will cause some PEI to fail to cross-link effectively, resulting in structural instability.

Therefore, based on the adsorption capacity and structural stability of PEI/ALG, the optimal contents of ALG, PEI, and the crosslinking agent in PEI/ALG are 0.2 g, 0.4 g, and 0.6 mL, respectively.

### 2.4. Influence of Zeta Potential and pH on the Adsorption Performance of Gel Beads

The results of the measurements of the zeta charge of ALG and PEI/ALG are shown in Figure 6c. Within the pH range of 2–12, the ALG surface is negatively charged. When PEI is loaded onto ALG, PEI/ALG is positively charged in the pH range from 2 to 5.79, because the amine groups of PEI are prone to protonation. As the pH changes from 5.79 to 11, the potential on the surface of PEI/ALG decreases, and the point of zero charge of PEI/ALG is 5.79. When the pH of the solution is higher than 5.79, a negative charge is formed at the surface of PEI/ALG.

When PEI/ALG is added to ultrapure water and Sb solutions with pH in the range of 3–6, the pH of the ultrapure water increases to 8.4, 9.1, 9.2, and 9.1 and that of the Sb solution increases to 5.7, 6.3, 6.5, and 6.6, respectively (Figure 6d). The reason for the increase in the pH of pure water is as follows. The dissociation constants (pKa) of the mannuronic acid and guluronic acid (carboxylic acids) in ALG are 3.38 and 3.65, respectively [30], and the pKa values of the-NH_2_, -NH-, and -N= on PEI are 4.5, 6.7, and 11.6, respectively [31]. When the pH_0_ of the solution is equal to 3, all of the -COOH and amino groups on PEI/ALG are protonated, resulting in a substantial elevation in the solution pH. At different pH values, the presence of Sb(III) is different. When the pH is less than 2, Sb(III) is dominated by positively charged Sb(OH)_2_^+^. Uncharged Sb(OH)_3_ is ubiquitous between pH 2 and 10.4. When pH is greater than 10.4, the dominant species are negatively charged H_2_SbO_3_^−^ or Sb(OH)_4_^−^ [32] (Table 1). The results of adsorption experiments showed that when the value of the Sb solution was between 3 and 5, the adsorption capacity remained between 144.14 ± 5.8 and 126.7 ± 9.1 mg/g. When pH_0_ increased to 6, the adsorption capacity of PEI/ALG increased significantly to 403.6 ± 9.1 mg/g. This is because with the increase in pH, the amino groups on the PEI chain are deprotonated, which not only increases the number of amino groups that can be used for coordination but also enhances their coordination strength, thus significantly improving the adsorption capacity of Sb(III) [10]. Our experiments show that the Sb solution generates white precipitate at pH 8 (Figure S2), so PEI/ALG causes the pH of the Sb solution to increase, resulting in the formation of Sb(OH)_3(s)_. This is manifested as an increase in the Sb removal effect. Since the formation of Sb(OH)_3(s)_ will bind to OH^−^ in the solution, the pH of the Sb solution after adsorption is lower than that of the pure aqueous solution. In addition to the formation of precipitation, the complexation of COOH and amino functional groups on PEI/ALG with Sb is also the main mechanism of Sb adsorption (refer to 2.2. adsorption mechanism).

In order to explore the relative contribution of precipitation and functional group adsorption, we set up two groups of solutions for experiments: the first group was pure water with pH = 6, and the second group was Sb(III) solution with initial concentration of 200 mg/L and pH = 6. First, the adsorbent was added to pure water at pH = 6, and the pH increased to 9.38 after shaking for 24 h. Based on this result, we also adjusted the Sb(III) solution with an initial concentration of 200 mg/L and pH = 6 to pH = 9.38 and oscillated for 24 h and found precipitation in the solution.

The results showed that in the control group without adsorbent, the concentration of Sb(III) solution decreased to 182.17 mg/L after 24 h, indicating that 17.83 mg/L of antimony was removed by precipitation (Figure S3). In the experimental group with PEI/ALG adsorbent, the concentration of Sb(III) further decreased to 16.76 mg/L at the same time. This indicates that although some of the removal of antimony can be attributed to the precipitation caused by the increase in pH, 82.7% of antimony is still removed mainly through functional group coordination and complexation, which plays a leading role in this adsorption process.

**Table 1:** The existing forms of antimony in antimony potassium tartrate in different pH solutions.

Reagent	Antimony Solution pH	Antimony Solution Concentration	Antimony Speciation	References
Antimony potassium tartrate	5	100–300 mg/L	pH = 0–2: Sb(OH)_2_^+^ pH = 2–10: Sb(OH)_3_ pH > 10: Sb(OH)_4_^−^	[10]
Potassium antimony tartrate hemihydrate (KSbO_4_H_4_O_7_·0.5H_2_O)	3	1000 mg/L	pH = 2–8: Sb(OH)_3_	[11]
Potassium antimony tartrate hemihydrate(C_8_H_4_K_2_O_12_Sb_2_·0.5H_2_O)	6	10–250 μg/L	pH = 2–10: Sb(OH)_3_	[33]
Potassium antimonyl tartrate trihydrate (C_8_H_4_K_2_O_12_Sb_2_·3H_2_O)	2–12	1000 mg/L	pH = 2: Sb(OH)_2_^+^ pH = 6–11: Sb(OH)_3_ pH > 11: Sb(OH)_4_^−^	[28]
Potassium antimonyl tartrate trihydrate (C_8_H_4_K_2_O_12_Sb_2_∙3H_2_O)	3–11	50 mg/L	pH = 2–11: Sb(OH)_3_	[34]
Potassium antimonyl tartrate trihydrate (C_8_H_4_K_2_O_12_Sb_2_.3H_2_O)	2–12	1000 mg/L	pH = 0–4: Sb(OH)_2_^+^ pH = 4–10: Sb(OH)_3_ pH > 11: Sb(OH)_4_^−^	[35]
Antimony potassium tartrate (C_8_H_10_K_2_O_15_Sb_2_)	1–11	10 mg/L	pH < 2: Sb(OH)_2_^+^ pH = 2–10: Sb(OH)_3_ pH > 10: H_2_SbO_3_^−^ and Sb(OH)_4_^−^	[36]
Potassium antimonyl tartrate trihydrate (C_8_H_4_K_2_O_12_Sb_2_.3H_2_O)	2–12	10–500 mg/L	pH < 2: Sb(OH)_2_^+^ pH = 2–10.4: Sb(OH)_3_ pH > 10.4: H_2_SbO_3_^−^ and Sb(OH)_4_^−^	[32]
Antimony potassium tartrate	2–10	100–800 mg/L	pH < 1.4: Sb(OH)_2_^+^ pH = 2–11: Sb(OH)_3_ pH > 11.8: Sb(OH)_4_^−^	[37]
Potassium antimony tartrate hemihydrate(C_8_H_4_K_2_O_12_Sb_2_·0.5H_2_O)	2–8	500 mg/L	pH = 2–10.4: Sb(OH)_3_	[38]
Antimony potassium tartrate (C_8_H_10_K_2_O_15_Sb_2_)	3–12	1000 mg/L	pH = 3–12: Sb(OH)_3_	[39]
Potassium antimonyl tartrate trihydrate (C_8_H_4_K_2_O_12_Sb_2_.3H_2_O)	2, 7, 11	1000 mg/L	pH = 2–11: Sb(OH)_3_	[40]
Antimony potassium tartrate	2–9	1000 mg/L	Sb(OH)_3_	[41]

### 2.5. Adsorption Kinetics Study

For Sb solution with a concentration of 150 mg/L, PEI/ALG reached adsorption equilibrium at 60 h (Figure 6e), and the adsorption capacities at 12 and 24 h were 88.3 mg/g and 242.1 mg/g, reaching 40% and 80% of its equilibrium adsorption capacity, respectively. For an Sb solution with a concentration of 300 mg/L, the adsorption amount of PEI/ALG for Sb(III) increased rapidly within 36 h and then increased gradually until it reached adsorption equilibrium at 72 h (Figure 6f). An increase in the Sb(III) concentration promoted a stronger adsorption driving force, resulting in a higher adsorption capacity of PEI/ALG at 300 mg/L. However, the adsorption rate decreased with time because when PEI/ALG came in contact with Sb(III), Sb(III) first occupied the active sites on the surface of PEI/ALG. With the passage of time, when the active sites were saturated, the accumulation of Sb(III) on the PEI/ALG surface repelled other Sb(III), preventing them from approaching the active sites, leading to a low Sb adsorption rate of PEI/ALG [15].

The determination of kinetic parameters is achieved using the pseudo-first and second-order models (Figure 6e,f) (Table S1). The correlation coefficients calculated using the pseudo-second-order model (0.991 and 0.998) were higher than those calculated using the pseudo-first-order model (0.985 and 0.973), indicating that the adsorption of Sb by PEI/ALG is predominantly governed by chemical adsorption. To improve the understanding of the rate-determining step in the adsorption mechanism of Sb(III) by PEI/ALG, the Elovich model was employed to facilitate the analysis of the adsorption kinetics data [11] (Figure 6g,h). Some Sb was adsorbed by PEI/ALG in the initial diffusion stage and then reached equilibrium in the second diffusion stage, and the diffusion rate (K_i_1) in the first stage was greater than that in the second stage (k_i_2). This is because Sb is rapidly adsorbed and saturated on the PEI/ALG surface through the film diffusion mechanism in the first stage, and the Sb content in the solution decreases with time, resulting in greater resistance to the diffusion of Sb(III) into the PEI/ALG particles in the second stage [42]. The Elovich model is based on the assumption that the adsorption process occurs on heterogeneous surfaces, and the adsorption process may be regulated by the diffusion factor and reaction rate [43]. The calculated α value is significantly higher when the Sb concentration is 150 mg/L than when the Sb concentration is 300 mg/L, indicating that PEI/ALG adsorbs more rapidly at lower concentrations [44].

### 2.6. Influence of Initial Sb Concentration and Adsorbent Dosage on the Adsorption Capacity of PEI/ALG

**Figure 7:**
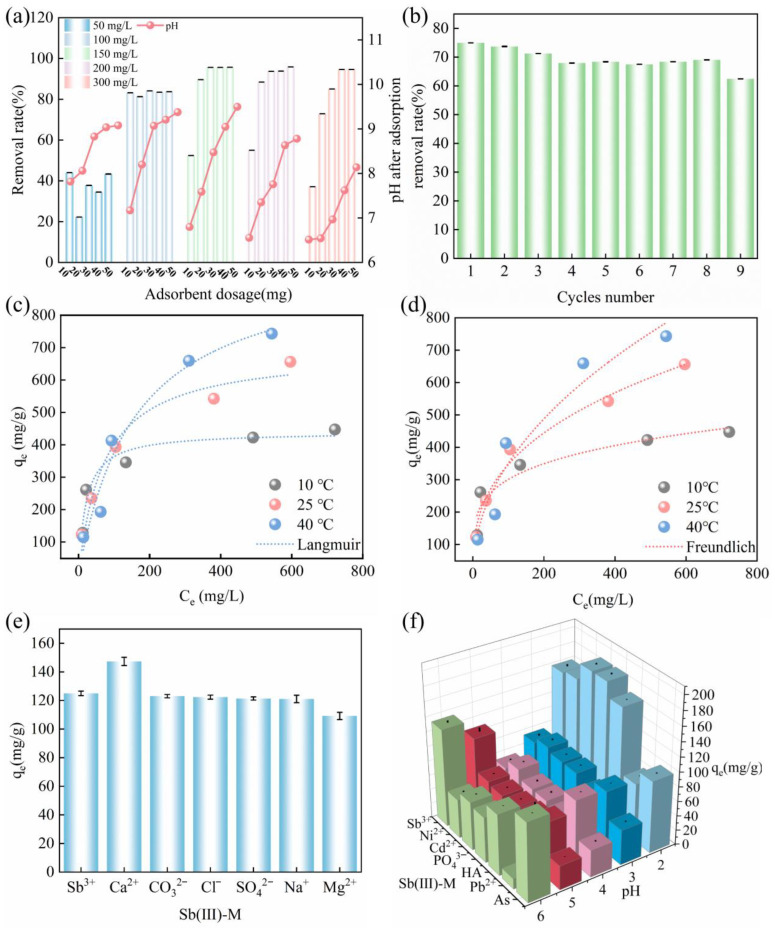
(**a**) Variation in the Sb(III) removal rate by PEI/ALG with the initial Sb(III) concentration ranging from 50 mg/L to 300 mg/L; (**b**) cyclical Sb adsorption by PEI/ALG; (**c**,**d**) isothermal adsorption curves for Sb adsorption by PEI; (**e**,**f**) influence of competitive ions on the adsorption capacity of PEI for Sb.

To study the Sb concentration that can be effectively treated by PEI/ALG at different dosages, the rates of Sb(III) removal by PEI/ALG at different dosages were investigated (Figure 7a). The rates of Sb(III) removal by PEI/ALG at different dosages (20, 30, 40, and 50 mg) at a low initial Sb(III) concentration (50 mg/L) were 34.5–43.5%, which were much lower than the Sb removal rates of 72.9–95.9% at high initial Sb(III) concentrations (100–300 mg/L), indicating that PEI/ALG was more suitable for Sb removal at high concentrations. When the PEI/ALG dosage was 10 mg, the Sb removal rate declined as the initial Sb concentration increased (100–300 mg/L). When the PEI/ALG dosage was 50 mg, the rate of Sb elimination increased with the increased initial Sb concentration (100–300 mg/L). The experimental results showed that PEI/ALG was more effective for the removal of high concentrations of Sb, but the Sb removal rate was also affected by the dosage of PEI/ALG.

At the same PEI/ALG dosage, after adsorption equilibrium, the pH of the solution decreased with increasing initial Sb content, indicating that PEI/ALG released H^+^ during Sb adsorption. At higher initial Sb concentrations, more Sb reacted with PEI/ALG, resulting in a lower pH value of the solution after adsorption. This is because at pH 6, the zeta potential on the surface of PEI is −7 mV, causing the deprotonation of -COOH. When Sb metal ions coordinate with existing -COO^−^, they consume the free -COO− in the solution. To maintain the dissociation equilibrium of carboxylate ions (Ka = [H^+^][-COO^−^]/[-COOH]), the equilibrium will shift to the right, meaning that more -COOH will dissociate and release H^+^ ions into the solution. Thus, the higher the initial Sb concentration, the greater the reaction between -COO^-^ and Sb on PEI/ALG, resulting in a higher concentration of H^+^ released, leading to lower solution pH.

### 2.7. Isothermal Adsorption Study

We further investigated the impact of two key variables on the adsorption capacity of PEI/ALG: the initial Sb concentration and the reaction temperature (Figure 7c,d). At 25 °C, in the concentration range of 100–1000 mg/L, as the Sb concentration increases, the adsorption capacity of PEI/ALG increased from 122.9 ± 3.9 mg/g to 656.2 ± 22.4 mg/g. As the concentration of Sb rises, there is concomitant enhancement in the driving force required to overcome the mass transfer resistance between PEI/ALG and the surface of the antimony solution, and the contact probability between Sb and the active sites of PEI/ALG also increases, resulting in the highest amount of Sb adsorbed by PEI/ALG. As the adsorption sites of PEI/ALG become saturated, the adsorption amount can no longer increase with increasing Sb concentration [15].

For the Sb concentrations ranging from 100 mg/L to 300 mg/L, the change in the temperature from 10 °C to 40 °C does not significantly affect the amount of Sb adsorbed by PEI/ALG. When the initial Sb concentration exceeds 300 mg/L, the Sb adsorption amount of PEI/ALG increases with increasing adsorption temperature and reaches 743.3 ± 23.0 mg/g. The increase in temperature can accelerate the movement of solute molecules in the system, increase the mass transfer rate and diffusion coefficient, and increase the adsorption capacity [45].In addition, the increase of q_max_ in the temperature range from 10 °C to 25 °C (from 447.5 to 656.2 mg/g) is much larger than that from 25 °C to 40 °C (from 656.2 to 743.3 mg/g), indicating that the effect of temperature rise on the adsorption capacity of 10–25 °C is significantly higher than that of 25–40 °C, because the endothermic reaction has a more significant effect on the initial stage of low temperature adsorption.

The isothermal adsorption data were analyzed and fitted using the Langmuir and Freundlich models [46]. The maximum adsorption capacities calculated using the Langmuir model at 10, 25, and 40 °C were 440.2, 690.9, and 978.1 mg/g, respectively (Table 2). Compared to the currently reported Sb adsorbents, PEI/ALG exhibits superior adsorption capacity (Table 3). A comparison of the determination coefficients R^2^ of the Langmuir and Freundlich models indicates that the Langmuir model provides a superior fit for the isothermal adsorption data, suggesting that the adsorption of Sb occurs via a monolayer mechanism. The Langmuir equilibrium constant K_L_ is in the range of 0–1, indicating that PEI/ALG is suitable for Sb(III) adsorption [47,48]. In the Freundlich adsorption model, the calculated 1/n is between 0 and 1, indicating that adsorption occurs easily; therefore, PEI/ALG is favorable for Sb(Ⅲ) adsorption [49].

In addition, the Dubinin-Radushkevich, Redlich-Peterson, Temkin, and Sips models also fit the isothermal adsorption data well (Table 2, Figure S4). Dubinin-Radushkevich is an empirical model that links the adsorption mechanism to the Gaussian energy distribution. The calculated average adsorption free energy E is used to determine that the adsorption mechanism is physical adsorption (E8 kJ/mol) [50,51].The average adsorption free energy E values of D-R at three temperatures were higher than 8 kJ/mol by fitting calculation. It can be speculated that chemical adsorption is dominant in the adsorption system studied. The Redlich-Peterson model can be regarded as a synthesis and promotion of the two classical models of Langmuir and Freundlich. The size of the R-P index (β) indicates the uniformity of the adsorption system. The β value obtained in the study tends to 1, indicating that the adsorption is uniform [50,52].The Temkin model effectively correlates the interaction between the adsorbent and the adsorbate and has a characteristic uniform binding energy distribution. In the case of ignoring the extreme concentration value, the model assumes that the adsorption heat decreases linearly with the surface coverage, and the constant of the Temkin model parameter B_T_ related to the change in adsorption energy is related to the adsorption heat [51]. The Sips model considers both saturation and heterogeneity. It is a combination of Langmuir and Freundlich isotherms. At low adsorbate concentration, it is similar to the Freundlich isotherm; at high concentrations, the monolayer adsorption characteristics similar to the Langmuir isotherm are described [53], and the index n of the Sips model is close to 1, indicating that the isotherm is more inclined to Langmuir [54].

**Table 3:** Comparison of adsorption performance of PEI/ALG and other reported adsorbents.

Adsorbents	Initial Sb Concentration (mg/L)	pH	Adsorbent Dosage (g/L)	Maximal Adsorption Capacity (mg/g)	References
HA@SA	--	5	2.7	195.7	[10]
GAD	10–220	6	8	7.67	[55]
PEI/BCFe	50–300	6	0.2	509.34	[27]
PPLB	75	3 ± 0.1	0.25	371.7	[11]
Mn-coated biochar	0–50	2–10	0.5	0.94	[56]
BMJR-nZVI	10–130	3–9	0.2	104.7	[3]
CH_1_BC	30–50	2–12	2	167.8	[28]
L_1_M_2_BO	0–50	7.0 ± 0.1	0.1	364.6	[57]
ZVIM	1–400	7	0.5	614.6	[58]
PEI/ALG	50–900	5	0.3	978.1	In this study

The thermodynamic distribution coefficients acquired at various temperatures were used to compute the thermodynamic parameters of the reactions involved in adsorption (ΔH°, ΔS°, and ΔG°). ΔH° is positive (13.25 kJ mol^−1^), indicating that the Sb(III) adsorption is an endothermic process, and the adsorption amount increases with temperature. ΔS° is positive (0.05 kJ k^−1^ mol^−1^), signifying an increase in the disorder of the solid–liquid interface upon adsorption of Sb(III) by PEI/ALG. ΔG° is negative (−2.25, −3.28, and −3.88 kJ mol^−1^), indicating that Sb(III) adsorption is spontaneous (Table S2) [49].

### 2.8. Adsorption Competition Experiment

CO_3_^2−^, PO_4_^3−^, Cl^−^, Ca^2+^, Mg^2+^, Cd^2+^, Ni^2+^, As, Pb^2+^ and HA coexist with Sb(III) in Sb-containing wastewater. The impact of competing ions on the adsorption capacity of PEI/ALG for Sb(III) must also be taken into account [1].

In this study, the presence of Ca^2+^ increased the amount of Sb adsorbed on PEI/ALG from 125.0 ± 1.6 to 147.3 ± 6.6 mg/g. The presence of Cl^−^, Na^+^, SO_4_^2−^, and CO_3_^2−^ did not affect the adsorption of Sb(III) by gel beads, while Mg^2+^ slightly inhibited the removal of Sb(III). We conducted a further investigation into the effects of H_2_PO_4_^−^/HPO_4_^2−^, Ni^2+^, Cd^2+^, Pb^2+^, As and HA on the adsorption of Sb on gel beads under pH 2–6. The results are presented in Figure 7e,f. When the pH is between 2 and 4, the ions and HA do not affect the Sb(III) adsorption by PEI/ALG. When the pH is between 5 and 6, the amount of adsorbed Sb(III) decreases significantly. When Sb(III) coexists with As or Pb, the adsorption capacity of PEI/ALG for Sb(III) decreases, indicating that there is a competitive adsorption between them.

In summary, in the binary competitive system, PEI/ALG showed good selectivity and anti-interference ability to Sb(III), especially against common anions (Cl^−^, SO_4_^2−^) and humic acid. However, the presence of phosphate and some heavy metal ions will significantly weaken its adsorption performance. This result shows that PEI/ALG is more suitable for the treatment of antimony-containing wastewater with low phosphate concentration. Its selectivity under the coexistence of multiple components in real wastewater still needs to be comprehensively evaluated in more complex systems.

### 2.9. Regeneration Experimental Research

The regeneration and reuse of adsorbents are important indicators of their economic feasibility. Nine adsorption–desorption experiments were performed for PEI/ALG. As shown in Figure S7, the PEI/ALG gel beads maintain their structural stability throughout the nine adsorption–desorption cycles. In addition, PEI/ALG retains 90–98% of the initial Sb removal rate in the first eight cycles, and the removal rate of Sb only decreases to 83% of the initial rate in the ninth cycle, which proves that PEI/ALG has good regeneration and adsorption capacity. The reason for the slight decrease in the PEI/ALG removal capacity is that some irreversible Sb(III) adsorption occupies the adsorption sites of PEI/ALG, and the desorption process of the NaOH solution reduces the number of available adsorption sites [11].

We further investigated the leaching of PEI and the internal residue of Sb during the cycle. About the PEI leaching problem: We measured the total nitrogen concentration in the first adsorption–desorption cycle solution. The data showed that the total nitrogen in the solution after adsorption was 12.6 mg/g, while in the subsequent NaOH desorption solution, the total nitrogen was 5.28 mg/g. This confirms that PEI does have a certain degree of leaching. However, the leaching amount is relatively low relative to the total amount of PEI loaded in the material. After multiple cycles, the crosslinking network tends to be stable, and the efficiency of subsequent cycles remains good (Figure S7), indicating that the main crosslinking structure is stable and PEI leaching will not continue to accumulate and lead to the collapse of the material structure.

About the internal residual problem of Sb: We measured the concentration of antimony in the solution of the first adsorption–desorption cycle and found that in a single cycle, the concentration of Sb adsorbed on the gel ball was about 74.25 mg/g, while the concentration of Sb desorbed with 0.1 M NaOH was only 3.56 mg/g. It shows that a large amount of Sb is retained inside the gel ball and is not released by the conventional desorption process. XPS surface analysis also supports this (Figure S4), which shows that the percentage of Sb atoms on the surface of the regenerated material is only slightly reduced from 2.61% to 2.41%, indicating that the surface Sb is effectively removed, but the internal Sb residue is significant. This part of Sb is likely to be fixed by strong coordination with multiple sites inside the gel network. The reason why the regeneration and utilization ability of the gel beads has been maintained well is that there are many active sites for Sb, and the future work should be to explore the desorption effect of the new desorption solution.

### 2.10. Dynamic Adsorption Experiment

**Figure 8:**
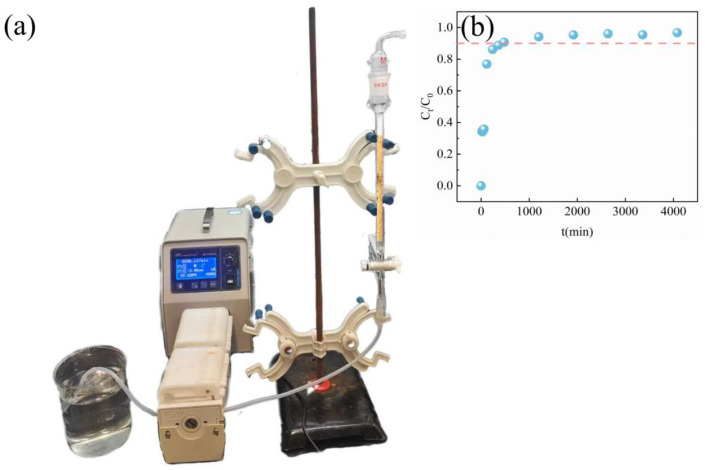
(**a**) Column adsorption of PEI/ALG for Sb(III). (**b**) Breakthrough curve of PEI/ALG to Sb(III).

In order to evaluate the practical application potential of PEI/ALG in Sb(III) solution [59], we carried out column adsorption experiments (Figure 8). When the concentration of Sb(III) solution was 100 mg/L and the flow rate was 1.5 mL/min, the penetration time of 0.6 g PEI/ALG to Sb(III) was 480 min, and the volume of treated Sb(III) solution was 1 L. The removal rate of Sb(III) by PEI/ALG can reach >95%.

## 3. Conclusions

In this study, three-dimensional porous network gel beads prepared from PEI and ALG were shown to effectively remove highly toxic Sb(III) from wastewater. The maximum experimental adsorption capacity of PEI/ALG for Sb(III) reached 743 mg/g, and the adsorption capability was unaffected by the Na^+^, Ca^2+^, or SO_4_^2−^ ions. After nine regeneration cycles, the gel beads still maintained an adsorption capacity retention of more than 83%, demonstrating stable structure and adsorption performance. DFT calculations and characterization results confirm that -NH_2_/-NH-/-N= groups dominate Sb(OH)_3_ adsorption through complexation, while the -COOH/-OH groups participate in adsorption through coordination and hydrogen bonding. It should be noted that this study also observed that the material is limited in practical applications: Sb(III) is prone to precipitate at higher pH conditions, which may interfere with the adsorption process; phosphate ions have a significant inhibitory effect on its adsorption performance; in addition, in the presence of coexisting cations/metalloids such as Ni, Cd, Pb, and As, the selectivity of the material to Sb(III) will be affected, and the recovery rate of antimony in the desorption solution after regeneration needs to be further improved. At the same time, this study mainly uses synthetic wastewater on a laboratory scale and has not yet evaluated the long-term performance and potential ecological toxicity of the material in actual complex water bodies. These are the directions that future research needs to focus on. Based on its good adsorption performance, stable structure, and reusability, PEI/ALG material shows the potential to be integrated into the actual sewage treatment system or to carry out pilot-scale research. In summary, this study not only clarified the adsorption nature of PEI/ALG on Sb(III) from the mechanism level but also provided theoretical support and technical reference for antimony pollution control and resource recovery and also pointed out the improvement direction for subsequent material optimization for complex actual water bodies.

## 4. Materials and Methods

### 4.1. Materials and Reagents

SA (CAS No. 9005-38-3, ultra-high viscosity type I, Viscosity (1% in H_2_O, 20 °C) = 5000 mPa·s, G:M = 1:1, molecular weight: 3.1 × 10^5^ Da) was purchased from Macklin Biochemical Technology Co., Ltd. (Shanghai, China). Comprehensive details regarding other materials and reagents can be found in the Supplementary Materials.

### 4.2. Preparation of Solution

The Sb(III) stock solution (500 mg/L) required for this experiment was prepared by dissolving C_8_H_4_K_2_O_12_Sb_2_·3H_2_O (solubility of 83,000 mg/L at 25 °C) in ultrapure water, and all test solutions were diluted by the stock solution.

### 4.3. Synthesis of Adsorbent

**Figure 9:**
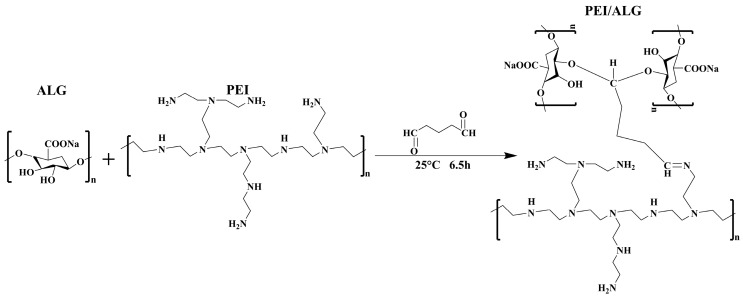
Reaction diagram of polyethyleneimine (PEI) aminated sodium alginate (ALG).

First, ALG, PEI, and pure water were stirred at 25 °C for 24 h, followed by the addition of glutaraldehyde (GLA) and continued stirring for 6.5 h. Then, gel solution was added into a 3% CaCl_2_ solution, thereby forming gel beads. Following a 12 h period, the gel beads were subjected to a series of washes with water, until the pH of the filtrate was the same as that of pure water. Finally, the gel beads were freeze-dried for 12 h and stored for future use. The results of the adsorption process demonstrated that the optimal PEI/ALG material was obtained using 0.2 g, 0.4 g, and 0.6 mL of ALG, PEI, and GLA, respectively (Figure 9).

### 4.4. Adsorption Experiments

Unless otherwise specified, the experimental conditions are as follows. PEI/ALG (30 mg) was added to the mixture (50 mL). The pH of the solution was equal to 6. The running time of the adsorption experiment was 48 h. The adsorption temperature was 25 °C.

#### 4.4.1. Effects of Different Influencing Factors on the Adsorption Behavior of PEI/ALG

The Sb(III) adsorption performance of the adsorbents at varying pH values was studied. To this end, an Sb solution with an original concentration of 200 mg/L was prepared, and the pH of the Sb(Ⅲ) solution was adjusted to 3, 4, 5, and 6. Then, PEI/ALG (30 mg) was added to the solutions with different pH values and the solutions were shaken for 24 h.

To investigate the effects of the temperature and Sb(Ⅲ) concentration on the adsorption performance of PEI/ALG, Sb(Ⅲ) solutions with initial concentrations of 50–300 mg/L were prepared, and adsorption experiments were performed at 298.15, 308.15, and 318.15 K.

A kinetics experiment was also conducted under Sb concentrations of 150 and 300 mg/L. The amount of added PEI/ALG was 30 mg, and the adsorption time was 0–7200 min.

The competitive adsorption experiment was conducted under the following conditions. Na^+^, Mg^2+^, Ca^2+^, SO_4_^2−^, SiO_3_^2−^, PO_4_^3−^, CO_3_^2−^, HA, Cd^2+^, Pb^2+^, As and Ni^+^ were mixed in pairs with the Sb(III) solution. The concentrations of Sb and the competing ions were 100 mg/L. The pH of the solution was 2–6.

The removal rate of PEI/ALG was studied as follows. Sb(III) solutions with initial concentrations of 50–300 mg/L were prepared. PEI/ALG (10, 20, 30, 40, and 50 mg) was added to Sb solutions (30 mL) with the concentrations of 50–300 mg/L to explore the removal rate of Sb(III).

#### 4.4.2. Regenerative Capacity Experiment

The initial concentration of Sb(III) solution was 200 mg/L. PEI/ALG after Sb(III) adsorption was desorbed with a 0.1 M NaOH solution (40 mL). Then, PEI/ALG was filtered and washed until the filtrate was neutral. After desorption, PEI/ALG was added to the Sb(III) solution for the next adsorption. The experiment was repeated 10 times.

#### 4.4.3. Dynamic Adsorption Experiment

The dynamic adsorption experiment was carried out in a chromatographic column with an inner diameter of 1.2 cm and a height of 20 cm. The mass of PEI/ALG gel beads filled in the chromatographic column was 0.6 g, and the height of the packed bed was 13 cm. The Sb(III) solution with an initial concentration of 100 mg/L was controlled by a peristaltic pump to enter the chromatographic column from bottom to top at a flow rate of 1.5 mL/min. Sb(III) solution was collected at different times until its concentration reached 90% of the initial concentration (depletion point).

#### 4.4.4. Adsorbent Characterization

The methods used for the characterization of the adsorbent are described in the Supplementary Materials.

### 4.5. DFT Calculations

The Gaussian 16, Rev. C01 software package was used to carry out the DFT calculations [60]. The hybrid PBE0 functional [61] and the D3 version of Grimme’s dispersion correction with Becke–Johnson damping (DFT-D3BJ) were used for all calculations. The SDD [62,63] basis set was utilized for Sb and the 6-31G(d) basis set was employed for other atoms for geometry optimization and frequency calculations. In single-point energy calculations, the SDD basis set was employed to model Sb, and the 6-311G(d,p) [64] set was used for the other atoms. The formula E_bind_ = E_complex_ − (E_partA_ + E_partB_) was used to calculate the interaction energy.

## Figures and Tables

**Table 2:** Isothermal adsorption parameters of PEI/ALG adsorbing Sb.

Adsorption Isotherm	T (°C)	Parameters			R^2^
Langmuir		q_max_ (mg/g)	K_L_ (L/mg)		
	10	440.2	0.048		0.92
	25	690.9	0.014		0.96
	40	978.1	0.006		0.95
Freundlich		K_F_ (L/mg)	1/n		
	10	112.2	0.214		0.87
	25	67.6	0.355		0.98
	40	39.4	0.474		0.92
Dubinin-Radushkevich		Q_DR_ (mg/g)	K_DR_ (mol^2^/kJ^2^)	E (kJ/mol)	
	10	405.09	3.3 × 10^−5^	123.09	0.90
	25	563.43	2.3 × 10^−4^	46.63	0.75
	40	728.89	7.6 × 10^−4^	25.65	0.93
Redlich-Peterson		K_RP_ (L/g)	α_RP_ (L/mg)	β	
	10	27.17	0.095	0.93	0.90
	25	28.56	0.22	0.74	0.98
	40	5.01	0.00103	1.25	0.93
Temkin		K_T_ (L/g)	B_T_ (J/mol)		
	10	1.06	63.34		0.91
	25	0.24	125.29		0.97
	40	0.095	183.61		0.90
Sips		Q_s_ (mg/g)	K_s_ (L/g)	n	
	10	464.55	0.04	0.8	0.89
	25	1142.32	0.0026	0.56	0.98
	40	919.25	0.0072	1.1	0.93

## Data Availability

The original contributions presented in this study are included in the article/Supplementary Materials. Further inquiries can be directed to the corresponding authors.

## Supplementary Materials

The following supporting information can be downloaded at: https://www.mdpi.com/article/10.3390/gels11121019/s1, Figure S1. The stress–strain curves of ALG, PEI and GLA gel spheres with different proportions under compression were obtained. (Maximum strain = 40%); Figure S2. The precipitation phenomenon of Sb(III) solution at pH 6.5,7,8; Figure S3. Control experiment without adsorbent; Figure S4. (a) Dubinin–Radushkevich (b) Redlich-Peterson (c) Temkin (d) Sips Isothermal model fitting; Figure S5. Total nitrogen content in adsorption solution and desorption solution.; Figure S6. XPS spectra of PEI/ALG after adsorption and desorption; Figure S7. Variation in Sb(III) removal rate by PEI/ALG with an initial Sb(III) concentration; Figure S8. The size of PEI/ALG (left) and ALG (right) gel spheres; Table S1: Dynamics parameters of PEI/ALG adsorbing Sb.; Table S2. Thermodynamic parameters of PEI/ALG adsorbing Sb.; Table S3. The content of each element after PEI/ALG adsorption and desorption.
