# Supplementary material for: Synergistic Effect of Aminated Sodium Alginate Composite Material on Selective Adsorption of Sb: Experimental and Density Functional Theory Study
Source: Gels. 2025 Dec 18;11(12):1019. doi: 10.3390/gels11121019 (PMC12732839; doi:10.3390/gels11121019)
Supplement: Supplementary file 1 [file gels-11-01019-s001.zip › gels-4009413-supplementary.pdf]

S4.1. Materials and reagents

PEI ($(CH_2CH_2NH)_n$, purity 99%), potassium antimonyl tartrate trihydrate ($C_8H_4K_2O_{12}Sb_2 \cdot 3H_2O$, purity 99%), glutaraldehyde (GLA) solution ($OHC(CH_2)_3CHO$, 25% in H_2O), sodium hydroxide (NaOH, purity 96%), calcium chloride ($CaCl_2$, purity 97%), sodium metasilicate nonahydrate ($Na_2SiO_3 \cdot 9H_2O$, analytically pure), HA ($C_{10}H_{19}O_7$, purity FA > 90%), and nickel chloride hexahydrate ($NiCl_2 \cdot 6H_2O$, AR 98%) were procured from Shanghai Aladdin Biochemical Technology Co., Ltd. ALG ($(C_6H_7NaO_6)_n$, ultra-high viscosity type I, 1% viscosity: 5000 mPa. s), and cadmium chloride ($CdCl_2$, purity 99.99%) were purchased from Shanghai Macklin Biochemical Technology Co., Ltd. Magnesium chloride ($MgCl_2 \cdot 6H_2O$, analytically pure), sodium chloride (NaCl, analytically pure), and anhydrous sodium sulfate (Na_2SO_4, analytically pure) were procured from Tianjin Zhiyuan Chemical Reagent Co., Ltd. Trisodium phosphate, dodecahydrate ($Na_3PO_4 \cdot 12H_2O$, purity 98%) was purchased from Yuanye Biotechnology Co., Ltd., and hydrochloric acid (HCl, analytically pure) was procured from Chengdu Kelong Co., Ltd.

S4.2. Adsorbent characterization

The preparation method for the characterized adsorbent was as follows. PEI/SA was added to 500 mL of a Sb(III) solution with an initial concentration of 100 mg/L and a pH of 6. The PEI/SA was shaken at 25 °C for 48 h and then filtered and dried for characterization.

Fourier transform infrared spectroscopy (FTIR) was utilised to obtain information regarding the functional groups present in the adsorbents (Bruker MPA and Tensor 27, Germany).X-ray photoelectron spectroscopy (XPS) was employed to examine the chemical composition of the surface components of the adsorbent (Thermo Fisher K-Alpha+, USA). X-ray diffraction (XRD) analysis was conducted to investigate the crystal structure of the adsorbent using Cu/Kα radiation (40 kV, 30 mA) at a scanning rate of 2°/min (Rigaku Smart Lab SE, Rigaku Corporation, Japan). Scanning electron microscopy (SEM) was utilised to facilitate the observation of the morphological features and size distribution of the adsorbent (Zeiss Sigma 300, Germany)., and the

specific surface area, pore volume and pore size distribution of the adsorbent were determined by employing a specific analyser (TriStar II 3flex, USA).

Table S1. Dynamics parameters of PEI/ALG adsorbing Sb

C_0	pseudo-first-order			pseudo-second-order		
	Q_e	k_1	R^2	Q_e	k_2	R^2
(mg/L)	(mg/g)	(1/min)		(mg/g)	(g/(mg·min))	
150	247.29	0.99	0.985	283.33	3.02×10^{-6}	0.991
300	456.56	3.98×10^{-4}	0.973	623.12	5.41×10^{-7}	0.998

Table S2. Thermodynamic parameters of PEI/ALG adsorbing Sb

ΔH^0 (kJ mol^{-1})	ΔS^0 (kJ K^{-1} mol^{-1})	ΔG^0 (kJ mol^{-1})		
		283 K	298 K	313 K
13.25	0.05	−2.25	−3.28	−3.88

Table S3. The content of each element after PEI/ALG adsorption and desorption.

Element content (%)	Adsorption	Desorption
C	58.68	59.44
O	28.35	27.39
N	9.88	10.4
Ca	0.48	0.36
Sb	2.61	2.41

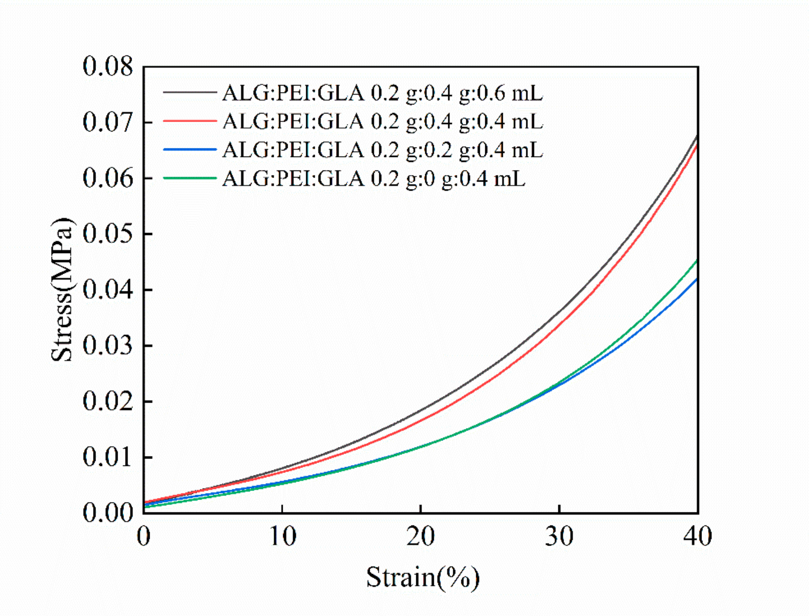

Figure S1. The stress-strain curves of ALG, PEI and GLA gel spheres with different proportions under compression were obtained. (Maximum strain = 40 %)

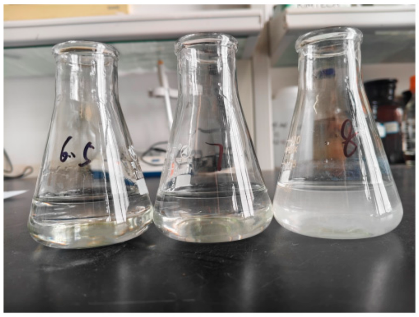

Figure S2. The precipitation phenomenon of Sb(III) solution at pH 6.5,7,8

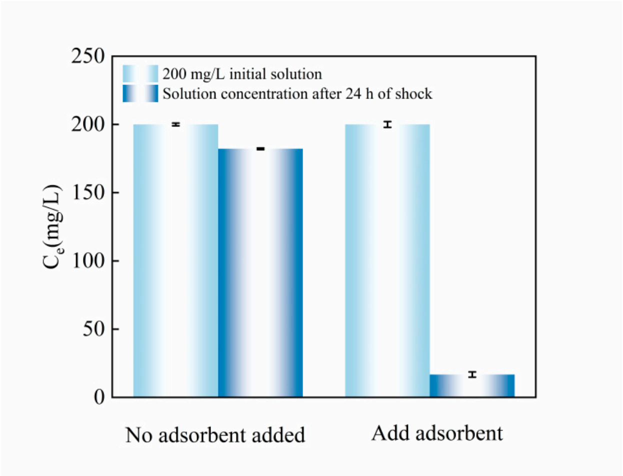

Figure S3. Control experiment without adsorbent

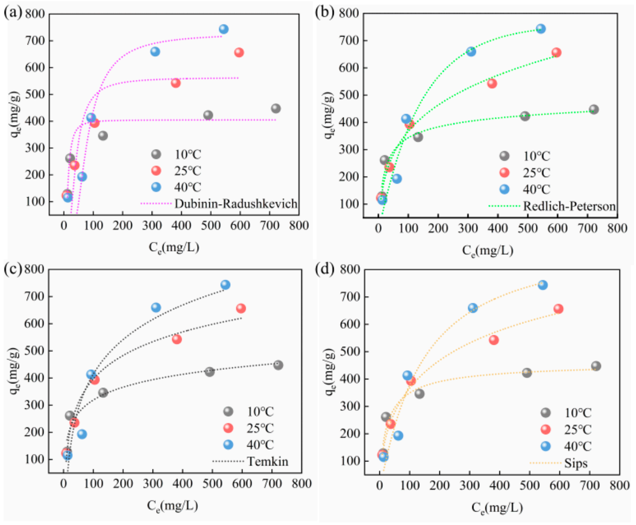

Figure S4. (a)Dubinin–Radushkevich (b)Redlich-Peterson (c) Temkin (d) SipsIsothermal model fitting

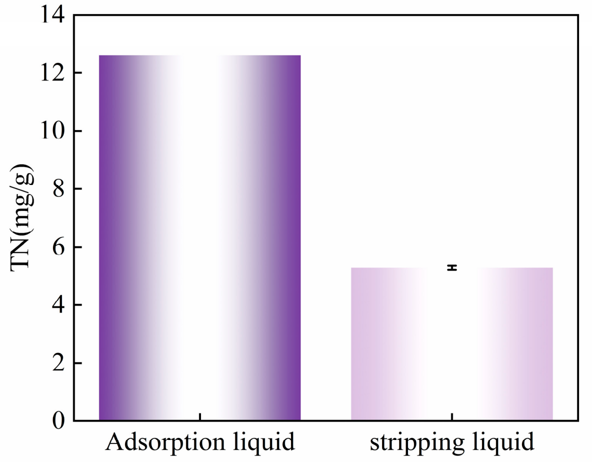

Figure S5. Total nitrogen content in adsorption solution and desorption solution

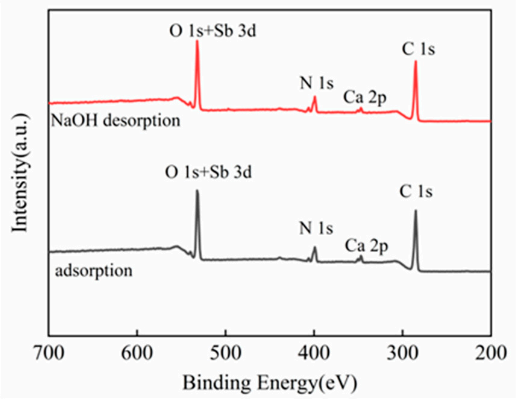

Figure S6. XPS spectra of PEI / ALG after adsorption and desorption

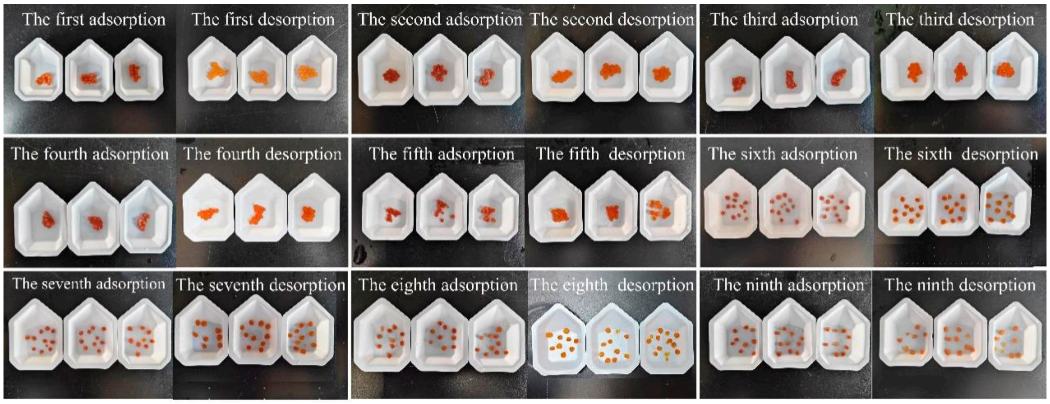

Figure S7. Variation in Sb(III) removal rate by PEI/ALG with an initial Sb(III) concentration

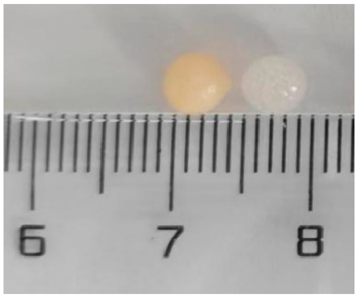

Figure S8. The size of PEI / ALG (left) and ALG (right) gel spheres.
